# Xylose-Assisted Glycolic Acid Overproduction in Engineered *Issatchenkia orientalis*

**DOI:** 10.3390/jof12100751

**Published:** 2026-10-07

**Authors:** Ran Ge, Xu Wang, Yufan Xing, Dongjie Yu, Lin Hu, Zixiang Xu, Qiaqing Wu, Dunming Zhu, Mingfeng Cao

**Affiliations:** 1 Key Laboratory for Synthetic Biotechnology of Xiamen City, Department of Chemical and Biochemical Engineering, College of Chemistry and Chemical Engineering, Xiamen University, Xiamen 361005, China; geran@stu.xmu.edu.cn (R.G.); xuwang2020@outlook.com (X.W.); yufan18888@163.com (Y.X.); yudongjie@stu.xmu.edu.cn (D.Y.); hulin@stu.xmu.edu.cn (L.H.); 2 Tianjin Institute of Industrial Biotechnology, Chinese Academy of Sciences, Tianjin 300308, China; zhu_dm@tib.cas.cn; 3 National Center of Technology Innovation for Synthetic Biology, National Engineering Research Center of Industrial Enzymes and Tianjin Engineering Research Center of Biocatalytic Technology, Tianjin 300308, China; 4 Key Laboratory of Engineering Biology for Low-Carbon Manufacturing, Tianjin 300308, China

**Keywords:** glycolic acid, *Issatchenkia orientalis*, metabolic engineering, glyoxylate cycle

## Abstract

Glycolic acid (GA) is an important platform chemical with applications in biodegradable polymers, cosmetics, and pharmaceutical synthesis; however, traditional petrochemical routes raise environmental concerns, and current microbial production platforms remain constrained by acid toxicity. In this study, systematic metabolic engineering combined with fermentation optimization was implemented to construct an efficient GA biosynthetic pathway in the acid-tolerant non-conventional yeast *Issatchenkia orientalis*. To redirect the metabolic flux of the glyoxylate cycle, the *Arabidopsis thaliana* glyoxylate reductase (*At*GLYR), with a catalytic efficiency approximately 74-fold higher than that of the native yeast enzyme, was heterologously expressed, coupled with the overexpression of endogenous isocitrate lyase (ICL) and the knockout of the competing malate synthase (MLS) gene. Furthermore, a heterologous xylose assimilation pathway was integrated into the engineered strain to rescue the growth deficiency caused by *mls* deletion. Subsequent fermentation optimization via an ethanol–xylose co-feeding strategy and calcium carbonate supplementation effectively further improved GA accumulation. Ultimately, the optimized engineered strain yielded a maximum GA titer of 712.42 mg/L in shake-flask fermentation. This work validates the potential of *I*. *orientalis* as a robust chassis microbe for sustainable GA biosynthesis and broadens the product portfolio of metabolic engineering applications for this yeast strain.

## 1. Introduction

**Figure 1:**
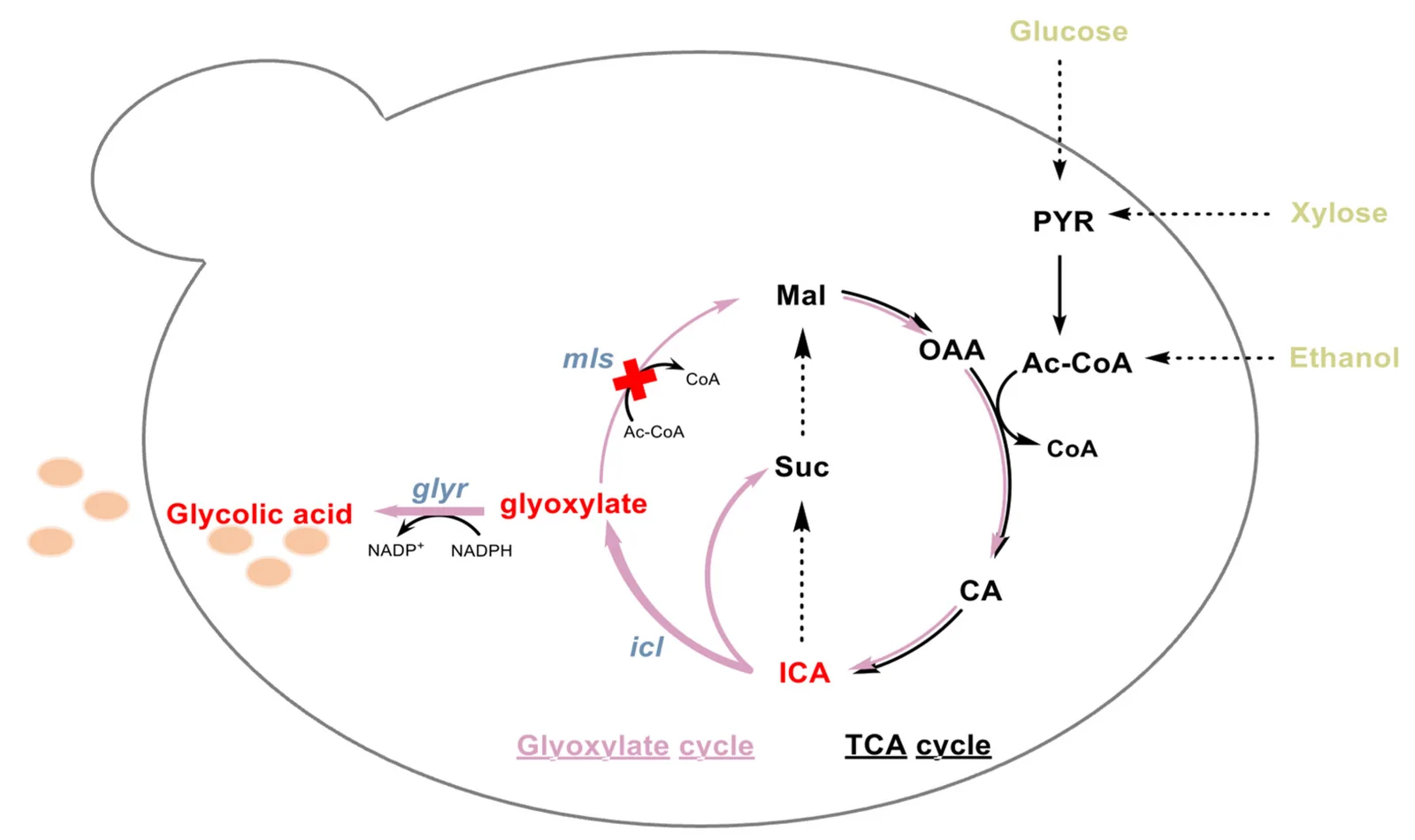
Schematic overview of the glyoxylate-cycle engineering strategy for GA biosynthesis in *I. orientalis*. Solid black arrows represent the reactions of the TCA cycle; solid pink arrows represent the reactions of the glyoxylate cycle, with the bold solid pink arrows indicating the overexpression of the corresponding genes; dashed arrows indicate the uptake of exogenous carbon sources (glucose, xylose, and ethanol); and the red cross indicates gene knockout. PYR: Pyruvate; Ac-CoA: Acetyl-CoA; CoA: Coenzyme A; CA: Citrate; ICA: Isocitrate; Suc: Succinate; Mal: Malate; OAA: Oxaloacetate; TCA cycle: Tricarboxylic acid cycle; icl: Isocitrate lyase; mls: Malate synthase; glyr: Glyoxylate reductase.

GA, the simplest α-hydroxycarboxylic acid, is extensively utilized in cosmetics for skin repair [1], in the chemical industry as a green cleaning agent [2], and as a direct monomer for the synthesis of biodegradable polyglycolic acid (PGA) and poly(lactic-co-glycolic acid) (PLGA) for biomedical and biodegradable plastic applications [3,4]. The global GA market is experiencing robust growth, driven primarily by increasing consumer demand for bio-based and environmentally sustainable products. As reported by Grand View Research, the market was valued at approximately USD 489.0 million in 2024 and is projected to expand at a compound annual growth rate (CAGR) of 7.1% through 2030 [5]. Currently, commercial GA production relies predominantly on petrochemical routes such as formaldehyde carbonylation [6] or oxalic acid hydrogenation [7], which involve toxic intermediates, harsh conditions, and considerable environmental burden. Microbial production of GA from renewable biomass has therefore emerged as an eco-friendly and sustainable alternative. The primary biosynthetic route involves redirecting the glyoxylate cycle: ICL cleaves isocitrate to generate glyoxylate, which is subsequently reduced to GA by glyoxylate reductase (GLYR) (Figure 1). *Escherichia coli* [8,9], *Saccharomyces cerevisiae*, and *Kluyveromyces lactis* [10] have been engineered for GA production via this pathway; however, the accumulation of GA severely drops the intracellular and extracellular pH, impairing cell growth and limiting the final titer.

*Issatchenkia orientalis* (syn. *Pichia kudriavzevii* or *Candida krusei*), a non-conventional diploid yeast, has emerged as a promising microbial chassis for organic acid production due to its intrinsic robustness under acidic conditions and elevated osmotic stress [11]. Recent advances in genetic tools [12], including the characterization of native promoters and terminators, the development of stabilized plasmid systems, and the implementation of CRISPR/Cas9-based landing pad strategies [13], have facilitated rational metabolic engineering of this host for the biosynthesis of lactic acid [14,15], citramalate [16], malic acid [17], succinic acid [18,19], itaconic acid [20], 5-aminolevulinic acid [21], and 3-hydroxypropionic acid [22]. Unlike conventional yeast hosts, *I. orientalis* is capable of maintaining efficient fermentation at low pH without alkali supplementation, which not only reduces base-neutralization costs but also mitigates contamination risks [10]. These attributes render it a particularly suitable platform for the production of acidic compounds such as GA.

Here, we present a metabolic engineering strategy to establish a robust GA production platform in *I. orientalis* (Figure 1). To redirect carbon flux toward GA, we introduced a high-affinity GLYR and overexpressed the native ICL, while deleting the competitive MLS to restrict glyoxylate consumption. Given that the glyoxylate cycle is subject to glucose-mediated catabolite repression, we further engineered a heterologous xylose utilization pathway. This modification allows the decoupling of growth from production via mixed feeding of xylose and ethanol, thereby facilitating the efficient conversion of lignocellulosic sugars into GA.

## 2. Materials and Methods

### 2.1. Strains, Media, and Materials

*Escherichia coli* DH5α was used for the maintenance and amplification of plasmids and was grown with antibiotics in LB medium. *Escherichia coli* BL21 was used for protein expression. IoCG-1 was propagated in YPD medium at 30 °C. Recombinant *I. orientalis* strains are cultured in synthetically complete deletion medium lacking uracil (SC-Ura). Table 1 summarizes the strains and plasmids used and constructed in this study.

### 2.2. Plasmids and Strains Construction

The codon optimized genes and primers are listed in the Tables S1 and S2. Plasmids are produced by Gibson assembly and Golden Gate assembly in *E. coli*. Promoters and terminators [12] were amplified from the genome of *I. orientalis.* The parental strain *I. orientalis* CICC 31129 was subjected to CRISPR-Cas9-mediated *ura3* deletion. A nourseothricin (NAT)-resistant Cas9 plasmid targeting the *ura3* locus was introduced, and a donor fragment with flanking homology arms was used for homology-directed repair, introducing a frameshift mutation. Transformants were selected on YPD agar with NAT (100 µg/mL). The Cas9 plasmid was cured by serial passaging (5 generations) in antibiotic-free YPD medium. The resulting URA auxotrophic strain was confirmed by PCR and sequencing and used as the starting strain IoCG-1 for downstream engineering. The successfully constructed plasmids were transformed into IoCG-1 via the lithium acetate method for strain construction [24]. The gene site-specific integration and knockout strains were constructed using the landing pad system [13].

### 2.3. Characterization of Glyoxylate Reductase Enzymatic Properties

Recombinant glyoxylate reductase harboring a His-tag was expressed in *Escherichia coli* BL21(DE3) and purified using Ni-NTA affinity chromatography. Enzyme activity was determined by monitoring the decrease in absorbance of NADPH at 340 nm. The standard reaction mixture (200 μL) contained 20 mM Tris-HCl (pH 7.5), 10 mM NADPH, 1–50 mM glyoxylate, and 2 μg of purified enzyme. The reaction was initiated by adding the substrate, and the absorbance was measured every 25 s for 4 min at 30 °C using a microplate reader. Kinetic parameters, including the Michaelis constant (*Km*) and maximum reaction velocity (*Vmax*), were calculated using the Lineweaver–Burk double-reciprocal plot method (Figure S1). It should be noted that the double-reciprocal transformation tends to overweight experimental errors, particularly at low substrate concentrations, and may therefore distort the estimation of kinetic parameters; accordingly, the kinetic parameters derived from the double-reciprocal plots should be regarded as comparative estimates rather than absolute values.

### 2.4. Fermentation Experiments

For shake flask fermentation, we inoculated individual colonies of *I. orientalis* strain into 10 mL of liquid SC-Ura medium containing 20 g/L carbon source and culture at 30 °C for 24 h. Then, we inoculated the cells into a 250 mL conical flask containing 30 mL SC-Ura liquid medium with different carbon sources at an initial OD_600_ of 0.2. Samples were taken every 12 h using a sampler to determine the cell density of the strain and the production of GA. All OD_600_ values reported in this article were measured after dilution: each culture sample was diluted 20–50-fold so that the measured absorbance fell within the linear range of the spectrophotometer (0.3–0.8), and the reported value is the dilution-corrected OD_600_. It should be noted that OD_600_ reflects total cell density and does not distinguish actively multiplying, resting, or dead cells; viable counts were not determined in this work.

In the fermentation supplemented with CaCO_3_, the seed culture was inoculated into 50 mL of SC-Ura medium to an initial OD_600_ of 0.2, along with a CaCO_3_ suspension at a final concentration of 3 g/L. The fermentation was carried out using mixed carbon sources of 10 g/L xylose and 10 g/L ethanol. Samples were taken every 12 h to monitor the pH and GA production. All shake-flask fermentations in this study were performed in two independent biological replicates per condition (*n* = 2).

### 2.5. Analytic Methods

Using Shimadzu LC-20AT HPLC (Shimadzu Corporation, Kyoto, Japan) and Aminex HPX-87H column (Bio-Rad Laboratories, Hercules, CA, USA) equipped with differential and ultraviolet detectors, the extracellular carbon source and GA concentration of the fermentation broth were measured, respectively. During differential detection, the column and detector were operated at 40 °C, using 0.5 mM H_2_SO_4_ as the mobile phase and a flow rate of 0.6 mL/min. During UV detection, the column and detector were operated at 50 °C, using 0.5 mM H_2_SO_4_ as the mobile phase with a flow rate of 0.5 mL/min and a detection wavelength of 210 nm.

### 2.6. qPCR Analysis

*I. orientalis* cells were seeded in SC-Ura medium and grown at 30 °C with constant shaking for 48 h. Total RNA was extracted using the RNase Mini Kit from Vazyme (Nanjing, China). DNase treatment of RNA was performed using Vazyme’s RNase Free DNase. cDNA synthesis was performed using Vazyme’s reverse transcription kit, and qPCR was performed using the SYBR mix from Biosharp (Hefei, China) following the manufacturer’s protocol. Primers for qPCR were designed using the IDT online tool (Primer Quest). The endogenous gene ALG9, which encodes mannosyltransferase, was used as an internal control. The expression of selected genes was normalized by ALG9 expression levels. Raw data were analyzed using Applied Biosystems’ QuantStudio Q3 real-time PCR (Waltham, MA, USA). qPCR analysis was performed with two biological duplicates.

## 3. Results

### 3.1. Evaluation of GA Tolerance in IoCG-1

**Figure 2:**
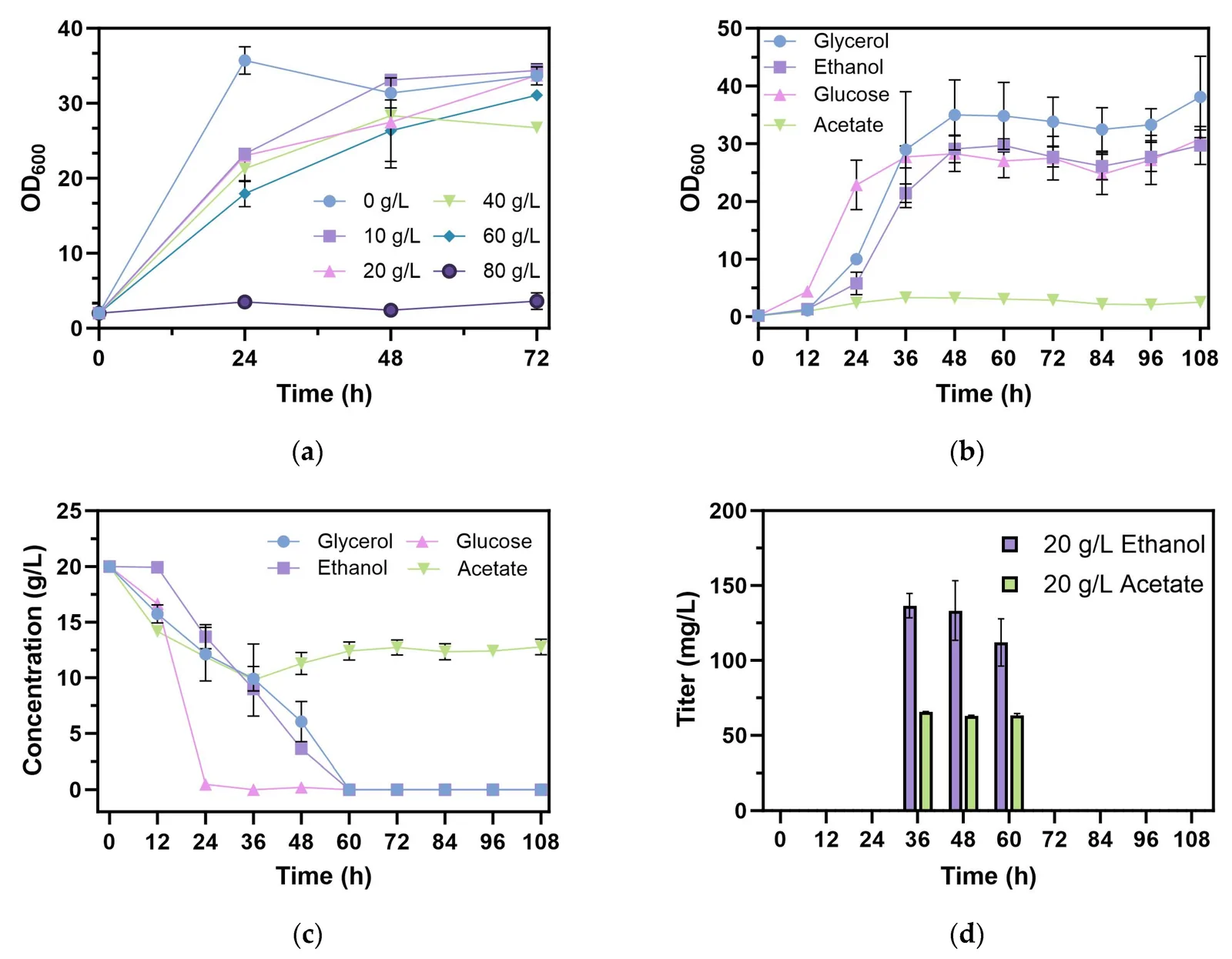
Physiological characterization of IoCG-1 and fermentation performance of strain Io-01 under different carbon source conditions. (**a**) GA tolerance of IoCG-1; (**b**) OD_600_ of strains; (**c**) carbon source consumption; (**d**) GA production.

The establishment of an efficient cell factory necessitates robust host strains capable of withstanding high concentrations of target products. We assessed the intrinsic tolerance of the IoCG-1 to GA. As shown in Figure 2a, IoCG-1 exhibited remarkable resistance and maintained a high cell density in the presence of 60 g/L GA. This confirmed the unique physiological advantage of *I. orientalis* in acidic environments and validated its selection as a superior host for GA biosynthesis.

### 3.2. Glyoxylate Reductase Selection and Initial GA-Producing Strain Construction

The reduction of glyoxylate to GA is directly catalyzed by GLYR. To achieve efficient GA biosynthesis, we systematically compared the kinetic parameters of two GLYR isoforms, namely the heterologous GLYR from *Arabidopsis thaliana* (*At*GLYR) [25] and the endogenous GLYR from *I*. *orientalis* (*Io*GLYR). As summarized in Table 2, *At*GLYR exhibited superior kinetic properties in vitro, with a higher apparent substrate affinity (*K*_m_ = 0.31 mM) and maximum reaction velocity (*V*_max_ = 1.488 mmol/min) compared to *Io*GLYR (*K*_m_ = 1.109 mM, *V*_max_ = 0.058 mmol/min). Notably, the overall catalytic efficiency of *At*GLYR was approximately 74-fold higher than that of *Io*GLYR.

Based on these kinetic advantages, *At*GLYR was selected for pathway construction and expressed using the stable plasmid pScARS/CEN-L, which contains a centromere-like (CEN-L) sequence previously shown to improve plasmid maintenance and stabilize heterologous gene expression in IoCG-1 [12]. The recombinant plasmid was subsequently transformed into the IoCG-1 to generate the initial GA-producing strain Io-01.

### 3.3. Effect of Carbon Source on Fermentation of Strain Io-01

To explore the optimal carbon source for GA synthesis, the fermentation performance of strain Io-01 was investigated in SC-Ura medium supplemented with 20 g/L glucose, glycerol, ethanol, or acetate, respectively. The cell density, substrate consumption, and GA production profiles under different carbon source conditions are presented in Figure 2. Strain Io-01 grew vigorously with glucose, glycerol, and ethanol as single carbon sources. Glucose was completely depleted within 24 h of fermentation, while glycerol and ethanol were fully consumed at 60 h. In contrast, growth on acetate as the sole carbon source was significantly stunted (Figure 2b,c). Despite the growth differences, GA accumulation was only detected in ethanol and acetate-supplemented media, with titers reaching 136 mg/L and 66 mg/L, respectively (Figure 2d). These results indicate that carbon source types profoundly affect intracellular precursor supply and thus determine GA biosynthesis capacity.

### 3.4. Metabolic Engineering to Optimize the GA Synthesis Pathway

**Figure 3:**
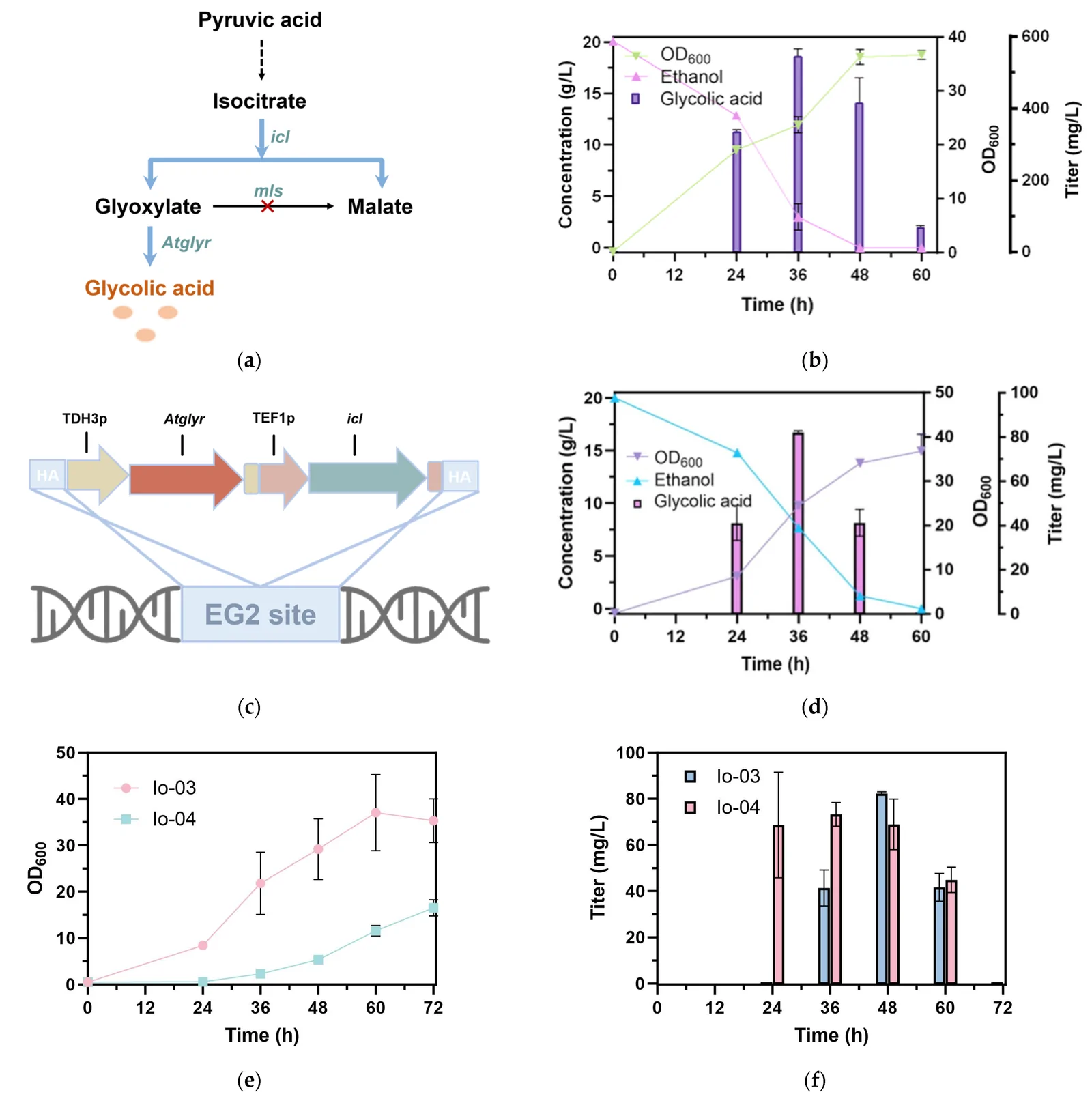
Metabolic engineering of the glyoxylate cycle to enhance GA production in *I. orientalis*: (**a**) Schematic of the glyoxylate-cycle engineering strategy; (**b**) GA production of strain Io-02 overexpressing icl on plasmid pScARS/CEN-L; (**c**) CRISPR/Cas9-mediated landing-pad integration of the AtGLYR–ICL pathway at the chromosomal EG2 site (strain Io-03); (**d**) fermentation performance of the genomically integrated strain Io-03; (**e**) cell density of the mls-disrupted strain Io-04 relative to Io-03; (**f**) GA titers of strains Io-03 and Io-04.

To improve intracellular glyoxylate accumulation and further enhance GA production, metabolic engineering strategies were implemented to optimize the glyoxylate cycle pathway (Figure 3a). Isocitrate lyase (ICL) catalyzes the cleavage of isocitrate to generate glyoxylate, which is the core step for intracellular glyoxylate synthesis. Therefore, endogenous *icl* was overexpressed to strengthen the supply of GA precursors.

Specifically, the *icl* gene was driven by the strong constitutive promoter TEF1p and co-expressed with *At*GLYR via the stable plasmid pScARS/CEN-L, yielding the engineered strain Io-02. Fermentation results showed that the GA titer of Io-02 reached 548.6 mg/L, which was 5.56-fold higher than that of the original strain Io-01 (Figure 3b), verifying that strengthening glyoxylate precursor supply effectively promotes GA synthesis.

Although plasmid-based expression enabled high GA production, the limited genetic cargo capacity of plasmid vectors constrained further pathway expansion and multigene engineering. To facilitate further metabolic engineering while ensuring genetic stability, a CRISPR/Cas9-mediated landing pad system was employed to site-specifically integrate the optimized pathway (*At*GLYR–ICL) into the chromosomal EG2 site, generating strain Io-03 (Figure 3c). As expected, genomic integration provided a more stable expression platform but resulted in a lower GA titer of approximately 82.3 mg/L relative to the plasmid-bearing strain Io-02 (Figure 3d). This reduction was likely attributable to the lower gene copy number associated with chromosomal integration compared with multicopy plasmid expression.

In the glyoxylate cycle, MLS catalyzes the condensation of glyoxylate with acetyl-CoA to form malate, thereby competing with GA biosynthesis for glyoxylate utilization. To redirect carbon flux toward GA production, the *mls* gene was disrupted in strain Io-03 via CRISPR/Cas9-mediated genome editing, yielding strain Io-04. Unexpectedly, *mls* deletion impaired cellular growth on ethanol, as evidenced by the significantly reduced growth rate of Io-04 relative to Io-03 (Figure 3e). Consequently, the reduced biomass formation negatively affected GA biosynthesis, resulting in a lower GA titer of 68.9 mg/L in strain Io-04 (Figure 3f).

### 3.5. Construction and Evaluation of Xylose-Metabolizing Modules

**Figure 4:**
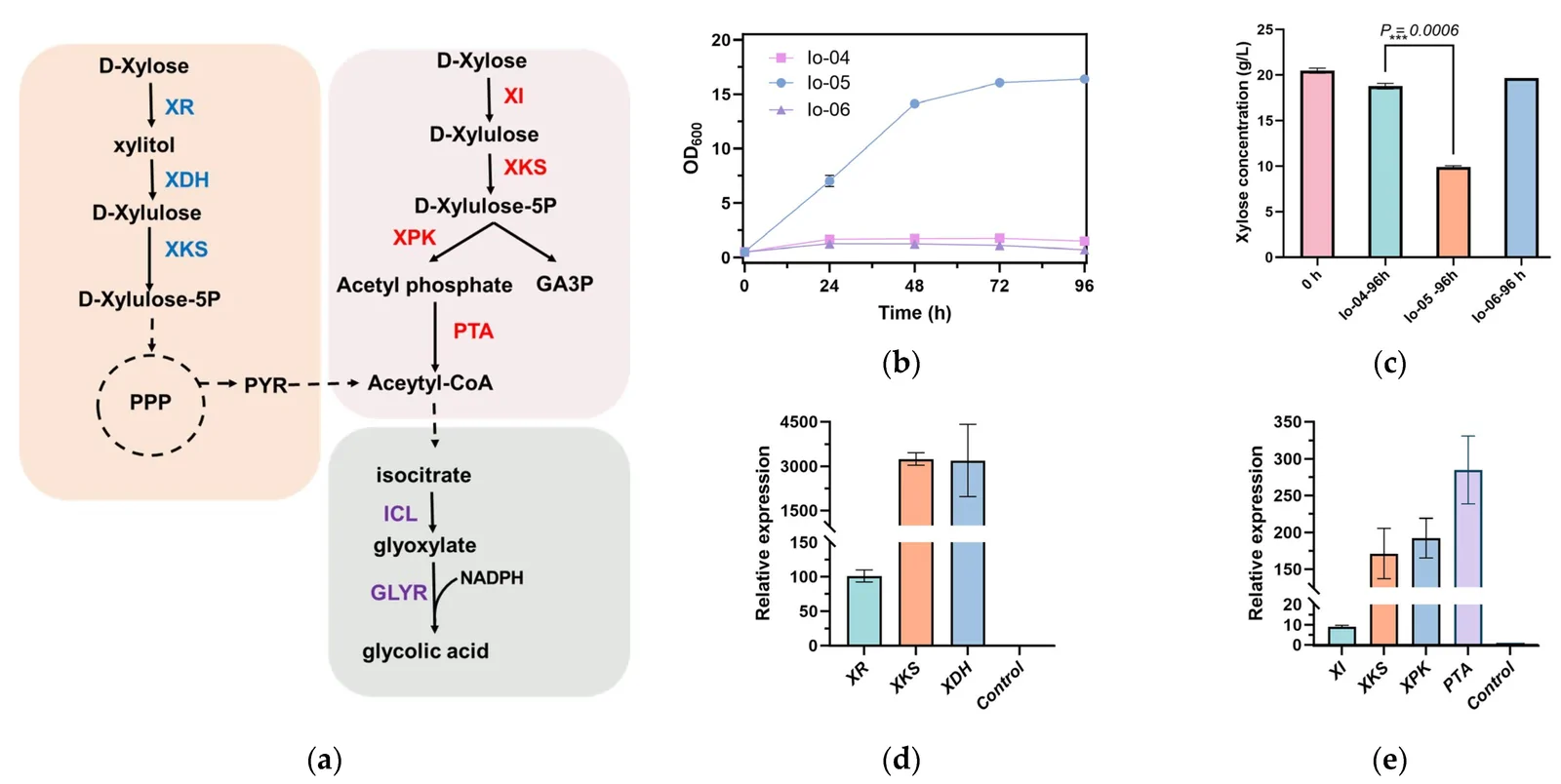
Construction and characterization of xylose-metabolizing engineered strains. (**a**) Schematic representation of xylose utilization pathways: the yellow is the XR/XDH pathway; the pink is XI pathway; the gray is the glyoxylic acid cycle. XR: xylose reductase; XDH: xylitol dehydrogenase; XI: xylose isomerase; XKS: xyloketokinase; XPK: xylose-5-phosphate ketotransferase; PTA: phosphate transacetylase. (**b**) cell density of strains Io-04, Io-05, and Io-06. (**c**) residual xylose concentration after 96 h fermentation. (**d**,**e**) relative transcription levels of xylose metabolism-related genes at 48 h.

The inactivation of malate synthase significantly compromises the ability of strain Io-04 to utilize ethanol for growth. Furthermore, the presence of glucose typically induces carbon catabolite repression (CCR), resulting in the downregulation of glyoxylate cycle genes. Conversely, previous studies have demonstrated that increasing xylose concentrations can activate the Snf1/Mig1 signaling pathway, thereby significantly upregulating key genes involved in gluconeogenesis, the glyoxylate cycle, and the TCA cycle [26]. To simultaneously overcome the growth defect and enhance GA biosynthesis, we constructed strains Io-05 and Io-06 by introducing the two well-established xylose utilization pathways previously validated in *Saccharomyces cerevisiae*, namely the oxidoreductive (XR–XDH–XKS) pathway [27] and the isomerase–phosphoketolase pathway (XI–XKS–XPK–PTA) [28], respectively (Figure 4a). Detailed information regarding the sources and sequences of the relevant plasmids and genes is provided in Table 1 and Table S1.

Among the tested strains (Io-04, Io-05, and Io-06), only Io-05 successfully utilized xylose for growth during fermentation. Both the parental strain Io-04 and the engineered strain Io-06 failed to proliferate when xylose was supplied as the sole carbon source (Figure 4b). Furthermore, xylose consumption by Io-05 reached approximately 10 g/L at 96 h, representing a significant increase relative to Io-04 (Figure 4c).

To investigate the expression profiles of the two xylose-utilization plasmids in the host and elucidate the mechanism underlying the failure of strain Io-06 to utilize xylose, we quantified the transcript levels of key genes at mid-log phase (48 h) by RT-qPCR. Following normalization against the reference gene *alg9*, the results showed that, although genes in the oxidoreductive (XR–XDH–XKS) pathway were detectably expressed, the relative expression of *xr* was approximately 30-fold lower than that of *xks* and *xdh* (Figure 4d). This low expression likely limits the rate of xylose conversion to xylulose and its subsequent entry into the pentose phosphate pathway (PPP), resulting in slow xylose utilization. In contrast, the *xi* gene in strain Io-06 was expressed at a low level, approximately 10-fold that of the reference gene (Figure 4e), which accounts for its failure to grow on xylose.

### 3.6. Xylose-Assisted GA Overproduction Through Mixed Carbon Metabolism

**Figure 5:**
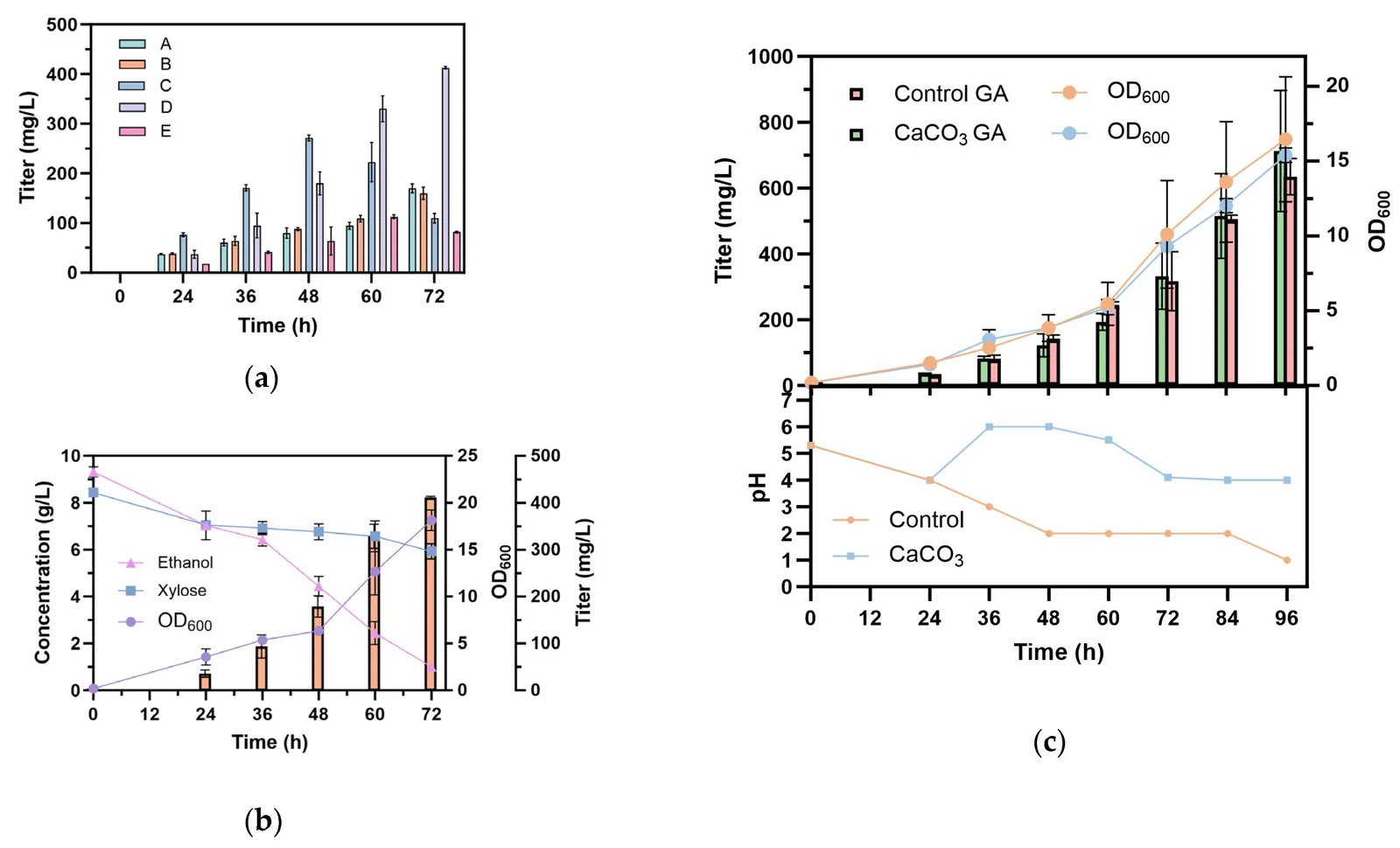
Optimization of fermentation strategies for GA production. (**a**) GA titers under different mixed carbon source conditions. A: 5 g/L acetic acid and 15 g/L xylose; B: 10 g/L acetic acid and 10 g/L xylose; C: 5 g/L ethanol and 15 g/L xylose; D: 10 g/L ethanol and 10 g/L xylose; E: 15 g/L ethanol and 5 g/L xylose. (**b**) Shake-flask fermentation performance using 10 g/L xylose and 10 g/L ethanol. (**c**) Effect of CaCO_3_ supplementation on strain fermentation performance and GA production.

Although strain Io-05 utilized xylose as the sole carbon source, no GA accumulation was observed under these conditions. Given that growth on ethanol or acetate induces glyoxylate cycle activity [29], mixed-carbon fermentation strategies combining xylose with ethanol or acetate were subsequently evaluated to enhance GA biosynthesis. Specifically, we designed six experimental groups with varying ratios of acetate/ethanol to xylose (Figure 5a). Among the combinations tested, Condition D (10 g/L ethanol and 10 g/L xylose) yielded the most favorable outcome, reaching a GA titer of 412.6 mg/L at 72 h, representing an approximately 7-fold increase compared to strain Io-04. Shake-flask profiling under this optimal condition revealed a sequential utilization pattern, in which strain Io-05 preferentially exhausted ethanol while consuming only approximately 2 g/L of xylose (Figure 5b).

### 3.7. CaCO_3_ Supplementation Improves Fermentation Performance

Since GA accumulation can acidify the fermentation environment and negatively affect cellular metabolism, buffering strategies were further investigated. We supplemented the fermentation medium with CaCO_3_ as an in situ buffering agent. While the pH of the control culture decreased to 2.0 within 48 h, the addition of CaCO_3_ effectively maintained the culture pH between 4.0 and 6.0 (Figure 5c). Strikingly, this relief of acid stress elevated the final GA titer to 712.42 mg/L at 96 h.

## 4. Discussion

Microbial production of GA has emerged as a sustainable alternative to petrochemical synthesis, yet its efficient biosynthesis remains constrained by limited precursor availability, competing metabolic pathways, insufficient cofactor supply, and poor tolerance to acidic fermentation environments. In this study, a systematic metabolic engineering strategy was established in *I. orientalis* by integrating heterologous enzyme selection, glyoxylate cycle rewiring, xylose metabolism engineering, and fermentation optimization (Figure 1). These efforts collectively enabled GA biosynthesis in a non-conventional acid-tolerant yeast.

Efficient reduction of glyoxylate to GA represents the terminal step of the biosynthetic pathway and therefore strongly influences pathway flux. In native hosts, endogenous enzymes frequently exhibit insufficient catalytic performance for supporting high-level metabolite production. Consistent with this notion, kinetic characterization demonstrated that *At*GLYR possessed substantially higher catalytic efficiency than *Io*GLYR, supporting its selection as the key catalytic component for pathway engineering. The enhanced performance of *At*GLYR likely reflects differences in physiological function and evolutionary adaptation. In *Arabidopsis thaliana*, NADPH-dependent glyoxylate reductases (*At*GLYR1/GLYR2) reduce potentially toxic glyoxylate to GA, primarily as a detoxification/bypass mechanism that can complement the canonical photorespiratory pathway [30]. Consequently, plant-derived enzymes have evolved high catalytic efficiency to accommodate substantial metabolic demand. Since intracellular glyoxylate levels in yeast are typically low and transient, there is likely limited evolutionary pressure to maintain highly efficient glyoxylate-reducing activity.

During fermentation on different carbon sources, glucose triggers carbon catabolite repression (CCR) [31], suppressing *icl* transcription and glyoxylate cycle activity, with isocitrate lyase activity remaining lower than that of isocitrate dehydrogenase in the TCA cycle. When glycerol is used as the sole carbon source, it is assimilated into central metabolism through the formation of dihydroxyacetone phosphate (DHAP). In principle, replenishment of TCA cycle intermediates under glycerol metabolism does not require the glyoxylate cycle, as oxaloacetate can be regenerated via pyruvate carboxylases (Pyc1/2) [32]. Consistent with this, the absence of glycolate accumulation suggests that the glyoxylate cycle functions as a nonessential auxiliary pathway with limited metabolic flux under glycerol conditions. In contrast, growth on ethanol or acetate, which serve as C2 carbon sources, requires the glyoxylate cycle to bypass the decarboxylation steps of the TCA cycle and enable the conversion of acetyl-CoA into C4 intermediates for biomass formation. Under these conditions, flux through the glyoxylate cycle is expected to increase, leading to greater intracellular glyoxylate availability. The elevated glyoxylate pool may consequently favor substrate channeling toward the highly efficient *At*GLYR, thereby enhancing GA formation and accumulation.

Because glyoxylate directly serves as the precursor for GA synthesis, increasing intracellular glyoxylate availability represented a rational engineering strategy. Overexpression of the endogenous *icl* gene substantially improved GA production, indicating that precursor generation through the glyoxylate cycle was one of the limiting factors in strain Io-01. By contrast, despite the expected benefit of blocking glyoxylate consumption, deletion of *mls* reduced GA production due to impaired growth on ethanol. This finding highlights an important metabolic trade-off during pathway engineering. Since ethanol assimilation in yeast strongly depends on the glyoxylate cycle for carbon conservation and replenishment of biosynthetic intermediates, disruption of MLS weakened the cellular capacity for efficient ethanol metabolism. Consequently, reduced biomass formation constrained overall metabolic productivity. Direct enumeration of viable cells, including CFU counting and viability staining, was not performed in this work (Section 2.4) and will be an important part of our follow-up studies as this process is scaled up.

To alleviate the growth burden caused by *mls* deletion, two heterologous xylose assimilation pathways were evaluated in the engineered Io-04. Among them, the XR–XDH–XKS pathway substantially outperformed the XI–XKS–XPK–PTA pathway with respect to both growth and xylose utilization. This difference may partly reflect the greater physiological compatibility of the oxidoreductive pathway with yeast metabolism, as many yeasts naturally employ reductase- and dehydrogenase-mediated sugar catabolism. Consequently, XR–XDH–XKS may integrate more efficiently into endogenous metabolic networks than heterologous xylose isomerase-based pathways. By contrast, although the XI pathway is employed by certain fungi and bacteria, its performance remained limited in *I. orientalis*, even when a fungal XI and strong promoters were used. Beyond potential transcriptional constraints, the pathway may suffer from poor enzyme compatibility, suboptimal catalytic activity, or inadequate intracellular folding, ultimately resulting in reduced xylose utilization efficiency [33].

No GA accumulation was observed when xylose was supplied as the sole carbon source. A possible explanation is that xylose-derived carbon primarily enters central metabolism via the pentose phosphate pathway and does not intrinsically require strong activation of the glyoxylate cycle. Consequently, glyoxylate cycle flux may remain insufficient to generate adequate intracellular glyoxylate for GA biosynthesis, although this regulatory constraint is likely less pronounced than the carbon catabolite repression observed under glucose conditions [34]. Interestingly, during co-fermentation with 10 g/L ethanol, strain Io-05 consumed only approximately 2 g/L xylose, compared with approximately 10 g/L when xylose served as the sole carbon source. Despite this limited xylose consumption, enhanced GA production was observed, suggesting that xylose contributes less as a primary carbon source and more as a complementary metabolic substrate. Co-utilization of xylose with ethanol or acetate may therefore integrate the metabolic advantages of both substrates, with C2 carbon sources enhancing glyoxylate precursor supply through the activation of the glyoxylate cycle, while xylose assimilation compensates for the depletion of C4 intermediates caused by *mls* deletion, thereby supporting cellular growth.

Supplementation with CaCO_3_ enhanced GA production and was associated with greater extracellular pH stability during fermentation. Although stabilization of culture pH may have contributed to performance, the effect of CaCO_3_ is unlikely to result solely from pH regulation, given the inherent acid tolerance of *I. orientalis*. On one hand, organic acid accumulation can compromise membrane integrity, enzyme activity, and nutrient transport, such that improved buffering may help sustain cellular metabolism during prolonged fermentation. On the other hand, CaCO_3_ provides a continuous source of HCO_3_^−^, which may relieve substrate limitation of Pyc1/2 and OAA formation [35]. Under ethanol conditions, increased OAA availability may enhance glyoxylate cycle flux, thereby promoting *At*GLYR-mediated conversion of glyoxylate to GA.

## 5. Conclusions

In this study, we established a systematic metabolic engineering strategy in *I. orientalis* for GA production. Comparative kinetic characterization identified the plant-derived *At*GLYR as a superior catalytic module for glyoxylate reduction, enabling efficient conversion of glyoxylate to GA. Carbon source evaluation revealed that ethanol and acetate, as C2 substrates, preferentially promoted GA production by activating glyoxylate cycle-dependent precursor generation. Subsequent pathway engineering through ICL overexpression significantly enhanced glyoxylate supply, whereas MLS deletion exposed a metabolic trade-off between precursor redirection and cellular growth. Furthermore, mixed-carbon fermentation using ethanol and xylose substantially improved GA production. Finally, CaCO_3_ supplementation further increased GA titer by stabilizing fermentation conditions and potentially enhancing precursor availability through bicarbonate-mediated metabolic support. Collectively, these strategies achieved a maximum GA titer of 712.4 mg/L under optimized mixed-carbon fermentation, demonstrating the potential of *I. orientalis* as a robust host for acid-tolerant bioproduction. This work provides a foundation for further scale-up and metabolic engineering of non-conventional yeasts for sustainable GA production.

## Figures and Tables

**Table 1:** List of plasmids and strains used in this study.

Plasmids/Strains	Description	Source
Plasmids		
pET28a	Kan^R^, T7p, T7t	lab collection
pET28a-IoGlyR	Kan^R^, T7p-IoGlyR-T7t	this study
pET28a-AtGlyR	Kan^R^, T7p-AtGlyR-T7t	this study
pScARS/CEN-L	Amp^R^, URA3, LEU2, CEN-0.8kb	Gifted from Zhao lab [12]
pScARS/CEN-L-*At*GlyR	pScARS/CEN-L with TDH3p-*At*GlyR-Tef1t	this study
pScARS/CEN-L-*At*GlyR-ICL	pScARS/CEN-L with TDH3p-*At*GlyR-Tef1t, Tef1p-ICL-3767t	this study
pScARS/CEN-L-XR-XDH-XKS	pScARS/CEN-L with HSP12p-*Ct*XR-PDC1t, GPMp-*Ct*XDH-MDH1t, INO1p-*Sc*XKS-PFK1t	this study
pScARS/CEN-L-XI-XKS-XPK-PTA	pScARS/CEN-L with HSP12p-*Op*XI-PDC1t, INO1p-*Sc*XKS-PFK1t, GPMp-*Lm*XPK-MDH1t, FBA1p-*Ck*PTA-5025t	this study
pVT36b-Cas9	Amp^R^, URA3, CRISPR/Cas9, sgRNA scaffold	Gifted from Zhao’s lab [23]
pVT36b-Cas9-EG2	pVT36b-Cas9 with EG2 sgRNA scaffold	this study
pVT36b-Cas9-MLS	pVT36b-Cas9 with MLS sgRNA scaffold	this study
Strains		
*E. coli* DH5α	Cloning host	lab collection
*Issatchenkia orientalis* CG-1 (IoCG-1)	Derived from *Issatchenkia orientalis* CICC 31129 with *ura3* knockout (Δ*ura*)	this study
Io-01	IoCG-1 with pScARS/CEN-L-*At*GlyR	this study
Io-02	IoCG-1 with pScARS/CEN-L-*At*GlyR-ICL	this study
Io-03	Integrate TDH3p-*At*GlyR-Tef1t and Tef1p-ICL-3767tGFP at the EG2 site of IoCG-1	this study
Io-04	Integrate TDH3p-*At*GlyR-Tef1t and Tef1p-ICL-3767tGFP at the EG2 site of IoCG-1, Δ*mls*	this study
Io-05	Io-04 containing plasmid pScARS/CEN-L-XR-XDH-XKS	this study
Io-06	Io-04 containing plasmid pScARS/CEN-L-XI-XKS-XPK-PTA	this study

**Table 2:** Kinetic parameters of glyoxylate reductase.

Protein	*K*_m_ (mM)	*V_max_* (mmol/min)	*k*_cat_ (s^−1^)	*k*_cat_/*K*_m_ (s^−1^ mM^−1^)
*At*GLYR	0.31	1.488	4.57	14.74
*Io*GLYR	1.109	0.058	0.22	0.20

## Data Availability

The original contributions presented in the study are included in the article/Supplementary Materials, further inquiries can be directed to the corresponding author.

## Supplementary Materials

The following supporting information can be downloaded at https://www.mdpi.com/article/10.3390/jof12100751/s1, Table S1: DNA sequences used in this study; Table S2: Primers used in this study; Figure S1: Double reciprocal plot of glyoxylate reductase on glyoxylic acid. (a) AtGLYR double re-ciprocal chart; (b) IoGLYR double reciprocal chart.

## References

[1] Liu Z., Ai J., Zhu Z., Huang L., Yi Z., Wu L., Xu Y., Zhu D., Li H., Yan Y. (2022). Chemical peeling with 35% glycolic acid for the treatment of disseminated facial verruca plana: A randomized, split-face, evaluator-blinded trial. Dermatol. Ther..

[2] Yadan L., Yan L., Liming C., Jiatian Z., Baojuan D., Yajun H., Chen L., Caoxing H., Chengrong Q., Shuangquan Y. (2021). High efficiency and clean separation of eucalyptus components by glycolic acid pretreatment. Bioresour. Technol..

[3] Susanne F., Marie W., Mats R., Anders A. (2011). The mechanisms of drug release in poly(lactic-co-glycolic acid)-based drug delivery systems—A review. Int. J. Pharm..

[4] Wang Y., Liu F., Chen J., Tse E.C.M., Shi R., Chen Y. (2025). Scale-up upcycling of waste polyethylene terephthalate plastics to biodegradable polyglycolic acid plastics. Nat. Commun..

[5] Team G.O.C.R. Glycolic Acid Market (2025–2030). https://www.grandviewresearch.com/industry-analysis/glycolic-acid-industry.

[6] Yu S., Lina H., Xu W., Heqin G., Litao J. (2026). Polymer-based coimmobilized -SO3H/-COOH catalyst for formaldehyde carbonylation to glycolic acid. Appl. Catal. A Gen..

[7] Hao L., Ren Q., Yang J., Luo L., Ren Y., Guo X., Zhou H., Xu M., Kong X., Li Z. (2023). Promoting electrocatalytic hydrogenation of oxalic acid to glycolic acid via an Al^3+^ ion adsorption strategy coupled with ethylene glycol oxidation. ACS Appl. Mater. Interfaces.

[8] Yong Y., Mengyao S., Di L., Feiyu F., Hongtao X., Fuping L., Changhao B., Xinna Z., Xueli Z. (2020). Construction of a carbon-conserving pathway for glycolate production by synergetic utilization of acetate and glucose in *Escherichia coli*. Metab. Eng..

[9] Yu D., Ning M., Kangjia Z., Yin M., Xuetuan W., Yunying Z. (2018). Balancing the carbon flux distributions between the TCA cycle and glyoxylate shunt to produce glycolate at high yield and titer in *Escherichia coli*. Metab. Eng..

[10] Koivistoinen O.M., Kuivanen J., Barth D., Turkia H., Pitkänen J.-P., Penttilä M., Richard P. (2013). Glycolic acid production in the engineered yeasts *Saccharomyces cerevisiae* and *Kluyveromyces lactis*. Microb. Cell Factories.

[11] Shih I.T., Zijun L., Vinh Gia T., Teresa Anne M., Huimin Z. (2025). *Issatchenkia orientalis* as a platform organism for cost-effective production of organic acids. Metab. Eng..

[12] Cao M., Fatma Z., Song X., Hsieh P.-H., Tran V.G., Lyon W.L., Sayadi M., Shao Z., Yoshikuni Y., Zhao H. (2020). A genetic toolbox for metabolic engineering of *Issatchenkia orientalis*. Metab. Eng..

[13] Fatma Z., Tan S.-I., Boob A.G., Zhao H. (2023). A landing pad system for multicopy gene integration in *Issatchenkia orientalis*. Metab. Eng..

[14] Park H.J., Bae J.-H., Ko H.-J., Lee S.-H., Sung B.H., Han J.-I., Sohn J.-H. (2018). Low-pH production of d-lactic acid using newly isolated acid tolerant yeast *Pichia kudriavzevii* NG7. Biotechnol. Bioeng..

[15] Lee Y.-G., Kang N.K., Kim C., Tran V.G., Cao M., Yoshikuni Y., Zhao H., Jin Y.-S. (2024). Self-buffering system for cost-effective production of lactic acid from glucose and xylose using acid-tolerant *Issatchenkia orientalis*. Bioresour. Technol..

[16] Wu Z.-Y., Sun W., Shen Y., Pratas J., Suthers P.F., Hsieh P.-H., Dwaraknath S., Rabinowitz J.D., Maranas C.D., Shao Z. (2023). Metabolic engineering of low-pH-tolerant non-model yeast, *Issatchenkia orientalis*, for production of citramalate. Metab. Eng. Commun..

[17] Xi Y., Xu H., Zhan T., Qin Y., Fan F., Zhang X. (2023). Metabolic engineering of the acid-tolerant yeast *Pichia kudriavzevii* for efficient L-malic acid production at low pH. Metab. Eng..

[18] Tran V.G., Mishra S., Bhagwat S.S., Shafaei S., Shen Y., Allen J.L., Crosly B.A., Tan S.-I., Fatma Z., Rabinowitz J.D. (2023). An end-to-end pipeline for succinic acid production at an industrially relevant scale using *Issatchenkia orientalis*. Nat. Commun..

[19] Tran V.G., Tan S.-I., Xu H., Weilandt D.R., Li X., Bhagwat S.S., Zhu Z., Guest J.S., Rabinowitz J.D., Zhao H. (2025). Decompartmentalization of the yeast mitochondrial metabolism to improve chemical production in *Issatchenkia orientalis*. Nat. Commun..

[20] Sun W., Vila-Santa A., Liu N., Prozorov T., Xie D., Faria N.T., Ferreira F.C., Mira N.P., Shao Z. (2020). Metabolic engineering of an acid-tolerant yeast strain *Pichia kudriavzevii* for itaconic acid production. Metab. Eng. Commun..

[21] Tan S.-I., Ng I.-S., Zhao H. (2025). Metabolic Engineering of Nonmodel yeast *Issatchenkia orientalis* SD108 for 5-aminolevulinic acid production. Biotechnol. Bioeng..

[22] Tan S.-I., Bhagwat S.S., Martin T.A., Suthers P.F., Tran V.G., Tang W., Fatma Z., Maranas C.D., Guest J.S., Zhao H. (2026). High yield production of 3-hydroxypropionic acid using *Issatchenkia orientalis*. Nat. Commun..

[23] Tran V.G., Cao M., Fatma Z., Song X., Zhao H. (2019). Development of a CRISPR/Cas9-based tool for gene deletion in *Issatchenkia orientalis*. mSphere.

[24] Gietz R.D., Schiestl R.H., Willems A.R., Woods R.A. (1995). Studies on the transformation of intact yeast cells by the LiAc/SS-DNA/PEG procedure. Yeast.

[25] Simpson J.P., Di Leo R., Dhanoa P.K., Allan W.L., Makhmoudova A., Clark S.M., Hoover G.J., Mullen R.T., Shelp B.J. (2008). Identification and characterization of a plastid-localized Arabidopsis glyoxylate reductase isoform: Comparison with a cytosolic isoform and implications for cellular redox homeostasis and aldehyde detoxification. J. Exp. Bot..

[26] Zeng W.-Y., Tang Y.-Q., Gou M., Xia Z.-Y., Kida K. (2016). Transcriptomes of a xylose-utilizing industrial flocculating *Saccharomyces cerevisiae* strain cultured in media containing different sugar sources. AMB Express.

[27] Zhang W., Zhang J., Zhao X., Zhang Z., He S., Bian X., Wang H., Zhang C., Lu W. (2024). Efficient production of ginsenoside Rh2 from xylose by remodeling metabolism in *Saccharomyces cerevisiae*. Chem. Eng. J..

[28] Huang M., Jin Z., Ni H., Zhang P., Li H., Liu J., Weng C., Jiang Z. (2024). Engineering the xylose metabolism of *Saccharomyces cerevisiae* for ethanol and single cell protein bioconversion. Biomass Bioenergy.

[29] Schöler A., Schüller H.J. (1993). Structure and regulation of the isocitrate lyase gene ICL1 from the yeast *Saccharomyces Cerevisiae*. Curr. Genet..

[30] Jiang X., Koenig A.M., Walker B.J., Hu J. (2025). A cytosolic glyoxylate shunt complements the canonical photorespiratory pathway in *Arabidopsis*. Nat. Commun..

[31] Gancedo J.M. (1998). Yeast carbon catabolite repression. Microbiol. Mol. Biol. Rev..

[32] Xiberras J., Klein M., Prosch C., Malubhoy Z., Nevoigt E. (2020). Anaplerotic reactions active during growth of *Saccharomyces cerevisiae* on glycerol. FEMS Yeast Res..

[33] Karhumaa K., Sanchez R.G., Hahn-Hägerdal B., Gorwa-Grauslund M.-F. (2007). Comparison of the xylose reductase-xylitol dehydrogenase and the xylose isomerase pathways for xylose fermentation by recombinant *Saccharomyces cerevisiae*. Microb. Cell Factories.

[34] Salusjärvi L., Kankainen M., Soliymani R., Pitkänen J.-P., Penttilä M., Ruohonen L. (2008). Regulation of xylose metabolism in recombinant *Saccharomyces cerevisiae*. Microb. Cell Factories.

[35] Malubhoy Z., Bahia F.M., de Valk S.C., de Hulster E., Rendulić T., Ortiz J.P.R., Xiberras J., Klein M., Mans R., Nevoigt E. (2022). Carbon dioxide fixation via production of succinic acid from glycerol in engineered *Saccharomyces cerevisiae*. Microb. Cell Factories.

