# Supplementary material for: Xylose-Assisted Glycolic Acid Overproduction in Engineered Issatchenkia orientalis
Source: J Fungi (Basel). 2026 Oct 7;12(10):751. doi: 10.3390/jof12100751 (PMC13705920; doi:10.3390/jof12100751)
Supplement: Supplementary file 1 [file jof-12-00751-s001.zip › jof-4592655-supplementary.pdf]

Supplementary Information

Xylose-Assisted Glycolic Acid Overproduction in Engineered *Issatchenkia orientalis*

Ran Ge [1,†], Xu Wang [1,†], Yufan Xing [1], Dongjie Yu [1], Lin Hu [1], Zixiang Xu [2,*], Qiaqing Wu [2,3,*], Dunming Zhu [2,3,4] and Mingfeng Cao [1,*]

[1] Key Laboratory for Synthetic Biotechnology of Xiamen City, Department of Chemical and Biochemical Engineering, College of Chemistry and Chemical Engineering, Xiamen University, Xiamen 361005, China; geran@stu.xmu.edu.cn (R.G.); xuwang2020@outlook.com (X.W.); yufan18888@163.com (Y.X.); yudongjie@stu.xmu.edu.cn (D.Y.); hulin@stu.xmu.edu.cn (L.H.)

[2] Tianjin Institute of Industrial Biotechnology, Chinese Academy of Sciences, Tianjin 300308, China; zhu_dm@tib.cas.cn

[3] National Center of Technology Innovation for Synthetic Biology, National Engineering Research Center of Industrial Enzymes and Tianjin Engineering Research Center of Biocatalytic Technology, Tianjin 300308, China

[4] Key Laboratory of Engineering Biology for Low-Carbon Manufacturing, Tianjin 300308, China

* Correspondence: xu_zx@tib.cas.cn (Z.X.); wu_qq@tib.cas.cn (Q.W.); mfcao@xmu.edu.cn (M.C.)

† These authors contributed equally to this work.

Table S1. DNA sequences used in this study

Gene name	DNA sequences
Atglyr	ATGGAAGTTGGTTTCTTAGGTTTAGGTATCATGGGTAAAGCTATGTCTA<br>TGAATTTGTTGAAAAATGGTTTTAAAGTTACTGTTTGGAATAGAACATT<br>ATCTAAATGTGATGAATTAGTTGAACATGGTGCTTCTGTTTGTGAATCT<br>CCAGCTGAAGTTATCAAAAAATGTAAATATACAATTGCTATGTTATCTG<br>ATCCATGTGCTGCTTTGTCAGTTGTTTTTGATAAAGGTGGTGTTTTAGA<br>ACAAATTTGTGAAGGTAAAGGTTATATTGATATGTCTACTGTTGATGCT<br>GAAACTTCTTTGAAAATTAATGAAGCTATTACTGGTAAAGGTGGTAGA<br>TTTGTTGAAGGTCCAGTATCTGGTTCTAAAAAACCAGCTGAAGATGGT<br>CAATTGATTATTTTAGCTGCTGGTGACAAAGCTTTGTTTGAAGAATCA<br>ATTCCAGCATTTGATGTTTTAGGTAAAAGATCTTTTTACTTAGGTCAAG<br>TTGGTAATGGTGCTAAAATGAAATTAATTGTTAATATGATTATGGGTTCT<br>ATGATGAATGCTTTCTCTGAAGGTTTGGTTTTGGCTGATAAATCAGGTT<br>TATCTAGTGATACATTATTAGATATTTTAGATTTGGGTGCTATGACAAAT<br>CCAATGTTTAAAGGTAAAGGTCCTTCTATGAATAAGTCTAGCTATCCAC<br>CTGCTTTTCCTTTGAAACATCAACAAAAAGATATGAGATTAGCTTTAG<br>CTTTGGGAGATGAAAATGCTGTTTCAATGCCAGTTGCTGCTGCTGCTA<br>ATGAAGCTTTTAAAAAAGCTAGATCTTTGGGTTTGGGTGACTTGGATT<br>TTTCAGCTGTTATTGAAGCTGTTAAATTTTCTAGAGAATAA

xr	ATGTCTACCACCCCAACCATCCCAACCATCAAGCTGAACTCTGGCTAC<br>GAGATGCCACTGGTGGGCTTCGGCTGTTGGAAGGTGAACAACGAGAC<br>CGCCGCCGATCAGATCTACAACGCCATCAAGACAGGCTACAGACTGTT<br>CGATGGCGCCGAGGATTACGGCAACGAGAAGGAGGTGGGCGAGGGC<br>ATCAACAGAGCCATCAAGGAGGGCCTGGTGAAGAGAGAGGAGCTGT<br>TCATCACCTCTAAGCTGTGGAACAACTTCCACGACCCAAAGAACGTG<br>GAGACCGCCCTGAACAAGACACTGAGCGACCTGAACCTGGATTACGT<br>GGATCTGTTCCTGATCCACTTCCCAATCGCCTTCAAGTTCGTGCCTATC<br>GAGGAGAAGTACCCACCAGGCTTCTACTGTGGCGATGGCGATAACTT<br>CCACTACGAGGATGTGCCACTGCTGGATACCTGGAAGGCCCTGGAGA<br>AGCTGGTGGAGGCCGGAAAGATCAAGAGCATCGGCATCTCTAACTTC<br>ACCGGCGCCCTGATCTACGATCTGATCAGAGGCGCCACCATCAAGCCA<br>GCCGTGCTGCAGATCGAGCACCACCCTTACCTGCAGCAGCCTAAGCT<br>GATCGAGTACGTGCAGAAGGCCGGCATCGCCATCACCGGCTACTCTTC<br>TTTCGGCCCACAGTCTTTCCTGGAGCTGGAGTCTAAGAGAGCCCTGA<br>ACACCCCAACCCTGTTCGAGCACGAGACCATCAAGTCTATCGCCGATA<br>AGCACGGCAAGTCTCCTGCCCAGGTGCTGCTGAGATGGGCCACCCAG<br>AGAAACATCGCCGTGATCCCAAAGTCTAACTCTCCTGAGAGACTGGC<br>CCAGAACCTGTCTGTGGTGGATTTCGATCTGACCCAGGAGGACCTGG<br>ACAACATCGCCAAGCTGGATATCGGCCTGAGATTCAACGATCCATGGG<br>ACTGGGATAACATCCCAATCTTCGTGTAA
xdh	ATGACAGCTAATCCTTCTCTGGTGCTGAATAAGGTGGATGATATTAGCT<br>TTGAAGAGTATGAAGCCCCTAAGCTGGAGTCTCCTAGAGATGTGATCG<br>TGGAGGTGAAAAAGACTGGCATCTGTGGCTCCGATATTCACTACTATG<br>CTCACGGAAGCATCGGCCCATTCATCCTGAGAAAGCCAATGGTGCTG<br>GGACATGAATCTGCCGGCGTGGTGTCAGCTGTGGGCAGTGAAGTGAC<br>TAATCTGAAGGTGGGCGATAGGGTGGCCATCGAGCCCGGAGTTCCCTC<br>TAGGTTTTCTGATGAGACTAAAAGCGGCCATTATCACCTGTGTCCACA<br>CATGAGCTTTGCAGCTACCCCACCTGTGAATCCCGATGAGCCAAATCC<br>ACAGGGCACCCTGTGCAAATATTACAGAGTGCCATGTGATTTCCTGTT<br>TAAACTGCCTGATCATGTGAGCCTGGAACTGGGAGCCATGGTGGAAC<br>CACTGACCGTGGGAGTCCATGGATGTAAGTTAGCTGATCTGAAGTTCG<br>GTGAGGATGTGGTGGTGTTTGGCGCCGGCCCTGTGGGGCTGCTGACA<br>GCCGCTGTTGCCAGAACAATTGGAGCCAAGCGCGTGATGGTGGTGGA<br>TATCTTCGATAACAAGCTGAAAATGGCTAAGGATATGGGAGCCGCAAC<br>ACACATCTTTAATTCTAAGACAGGAGGAGATTACCAGGATCTGATCAA<br>GTCTTTTGATGGCGTGCAGCCATCTGTGGTGCTGGAATGTTCTGGCGC<br>CCAGCCATGTATCTATATGGGTGTGAAGATCCTGAAGGCTGGAGGTAG<br>GTTTGTGCAGATCGGAAATGCTGGCGGAGATGTGAACTTCCCAATCGC<br>TGATTTTTCTACAAGAGAGCTGGCCCTGTACGGATCCTTTAGATACGG<br>CTATGGAGATTACCAGACATCCATTGATATTCTGGACAGAAACTACGTG<br>AATGGCAAAGATAAAGCTCCAATCAACTTTGAGCTGCTGATTACCCAT<br>AGATTCAAATTCAAGGATGCTATTAAGGCCTATGATCTGGTGAGAGCC<br>GGCAACGGCGCCGTGAAGTGTCTGATCGATGGCCCTGAGTAA

xks	ATGTTGTGTTCAGTAATTCAGAGACAGACAAGAGAGGTTTCCAACAC<br>AATGTCTTTAGACTCATACTATCTTGGGTTTGATCTTTCGACCCAACAA<br>CTGAAATGTCTCGCCATTAACCAGGACCTAAAAATTGTCCATTCAGAA<br>ACAGTGGAATTTGAAAAGGATCTTCCGCATTATCACACAAAGAAGGG<br>TGTCTATATACACGGCGACACTATCGAATGTCCCGTAGCCATGTGGTTA<br>GAGGCTCTAGATCTGGTTCTCTCGAAATATCGCGAGGCTAAATTTCCAT<br>TGAACAAAGTTATGGCCGTCTCAGGGTCCTGCCAGCAGCACGGGTCT<br>GTCTACTGGTCCTCCCAAGCCGAATCTCTGTTAGAGCAATTGAATAAG<br>AAACCGGAAAAAGATTTATTGCACTACGTGAGCTCTGTAGCATTTGCA<br>AGGCAAACCGCCCCCAATTGGCAAGACCACAGTACTGCAAAGCAATG<br>TCAAGAGTTTGAAGAGTGCATAGGTGGGCCTGAAAAAATGGCTCAAT<br>TAACAGGGTCCAGAGCCCATTTTAGATTTACTGGTCCTCAAATTCTGA<br>AAATTGCACAATTAGAACCAGAAGCTTACGAAAAAACAAAGACCATT<br>TCTTTAGTGTCTAATTTTTTGACTTCTATCTTAGTGGGCCATCTTGTTGA<br>ATTAGAGGAGGCAGATGCCTGTGGTATGAACCTTTATGATATACGTGA<br>AAGAAAATTCAGTGATGAGCTACTACATCTAATTGATAGTTCTTCTAAG<br>GATAAAACTATCAGACAAAAATTAATGAGAGCACCCATGAAAAATTTG<br>ATAGCGGGTACCATCTGTAAATATTTTATTGAGAAGTACGGTTTCAATA<br>CAAACTGCAAGGTCTCTCCCATGACTGGGGATAATTTAGCCACTATATG<br>TTCTTTACCCCTGCGGAAGAATGACGTTCTCGTTTCCCTAGGAACAAG<br>TACTACAGTTCTTCTGGTCACCGATAAGTATCACCCCTCTCCGAACTAT<br>CATCTTTTCATTCATCCAACTCTGCCAAACCATTATATGGGTATGATTTG<br>TTATTGTAATGGTTCTTTGGCAAGGGAGAGGATAAGAGACGAGTTAAA<br>CAAAGAACGGGAAAATAATTATGAGAAGACTAACGATTGGACTCTTTT<br>TAATCAAGCTGTGCTAGATGACTCAGAAAGTAGTGAAAATGAATTAGG<br>TGTATATTTTCCTCTGGGGGAGATCGTTCCTAGCGTAAAAGCCATAAAC<br>AAAAGGGTTATCTTCAATCCAAAAACGGGTATGATTGAAAGAGAGGT<br>GGCCAAGTTCAAAGACAAGAGGCACGATGCCAAAAATATTGTAGAAT<br>CACAGGCTTTAAGTTGCAGGGTAAGAATATCTCCCCTGCTTTCGGATT<br>CAAACGCAAGCTCACAACAGAGACTGAACGAAGATACAATCGTGAA<br>GTTTGATTACGATGAATCTCCGCTGCGGGACTACCTAAATAAAAGGCC<br>AGAAAGGACTTTTTTTGTAGGTGGGGCTTCTAAAAACGATGCTATTGT<br>GAAGAAGTTTGCTCAAGTCATTGGTGCTACAAAGGGTAATTTTAGGCT<br>AGAAACACCAAACTCATGTGCCCTTGGTGGTTGTTATAAGGCCATGTG<br>GTCATTGTTATATGACTCTAATAAAATTGCAGTTCCTTTTGATAAATTTC<br>TGAATGACAATTTTCCATGGCATGTAATGGAAAGCATATCCGATGTGGA<br>TAATGAAAATTGGGATCGCTATAATTCCAAGATTGTCCCCTTAAGCGAA<br>CTGGAAAAGACTCTCATCTAA
xi	ATGACAAAGGAATACTTCCCAACTATTGGTAAAATTAGATTCGAAGGT<br>AAGGATTCTAAGAATCCAATGGCTTTTCATTATTACGATGCTGAAAAA<br>GAAGTCATGGGTAAAAAAATGAAGGATTGGTTGAGATTTGCTATGGCT<br>TGGTGGCATACTTTGTGTGCTGATGGTGCTGATCAATTTGGTGTTGGTA<br>CTAAATCTTTTCCATGGAATGAAGGTACAGATCCAATTGCTATTGCTAA<br>ACAAAAAGTTGATGCTGGTTTTGAAATCATGACAAAATTGGGTATTGA

	GCATTATTGTTTCCATGATGTTGATTTGGTTTCTGAAGGTAATTCTATTG<br>AAGAATACGAATCTAACCTGAAACAAGTTGTTGCTTATTTGAAACAAA<br>AGCAACAAGAAACAGGTATTAAGTTGTTATGGTCTACTGCTAATGTTT<br>TTGGTAATCCAAGATATATGAACGGTGCTTCTACTAATCCAGATTTTGA<br>TGTTGTTGCTAGAGCTATTGTTCAAATTAAAAACGCTATGGATGCTGGT<br>ATTGAATTGGGTGCTGAAAATTATGTTTTCTGGGGTGGTAGAGAAGGT<br>TATATGTCTTTGTTGAATACAGATCAAAAGAGAGAAAAGGAACATATG<br>GCTACTATGTTAACTATGGCTAGAGATTATGCTAGATCTAAAGGTTTTA<br>AGGGTACTTTTTTGATCGAACCAAAACCAATGGAACCAACAAAACAT<br>CAATATGATGTTGATACTGAGACTGTTATTGGTTTTTTGAGAGCTCATA<br>ATTTGGATAAAGACTTTAAGGTTAACATCGAAGTTAACCATGCTACATT<br>AGCTGGTCATACATTTGAACATGAATTGGCTTGTGCTGTTGATGCTGG<br>AATGTTAGGTTCTATTGATGCTAATAGAGGTGATTATCAAAACGGTTGG<br>GATACAGATCAATTTCCAATTGATCAATACGAATTGGTTCAAGCTTGGA<br>TGGAAATTATTAGAGGTGGTGGTTTTGTTACAGGTGGTACAAATTTTG<br>ATGCTAAAACAAGAAGAAACTCCACTGATTTAGAAGATATTATCATCG<br>CTCATATCTCTGGTATGGATGCTATGGCTAGAGCTTTAGAAAATGCTGC<br>TAAATTATTGCAAGAGTCTCCATATTGTAACATGAAAAAAGAAAGGTA<br>CGCTTCTTTTGATTCTGGTATTGGTAAAGATTTCGAAGATGGTAAATTG<br>ACTTTGGAACAAGTTTATGAATACGGTAAAAAGAACGGTGAACCAAA<br>AGTTACTTCTGGTAAACAAGAATTGTACGAAGCTATTGTTGCTATGTAT<br>CAATAA
xpk	ATGAACATTGATTCTACCGACTACCTAAACAACCTAGATGCTTACTGG<br>AGAGCTACCAACTACTTGTCCGTCGGTCAATTATACTTGTTAGACAAC<br>CCATTATTGAAGGAGAAGTTGACTGCTGAACAAGTTAAGATTCACCCA<br>ATTGGTCACTGGGGTACTATCCCATCTCAAAACTTCATCTACGCTCACC<br>TAAACCGTGCCATTAACAAGTTCAACTTGAACATGTTCTACATTGAAG<br>GTCCAGGTCACGGTGGTCAAGTAATGATTTCCAACGCTTACTTGGACG<br>GTTCTTACACGGAAGCCTTCCCAGAAATCACTCAAGACGAAGCTGGT<br>ATGCAAAAGATGTTTAAGAGATTTTCTTTCCCAGGTGGTGTTGCTAGC<br>CACGCCGACCCAAAGGTTCCAGGTTCTATCCACGAAGGTGGTGCTTT<br>GGGATATTCCATCTTGCACGGCGCTGGTGCTGTCTTGGATAACCCTGA<br>CTTGATTGCTGCTGTCGTCGTTGGTGATGGTGAAGCTGAAACTGCTCC<br>ATTGGCCACTTCCTGGCACGTTAACAAATTCTTGAACCCAAAGAACGA<br>CGGTACTGTTTTGTTGATCTTGAACTTGAACGGTTTCAAGATCGCTAA<br>CCCTACTGTTTTGTCCAGAGAATCTGACGAAACCTTGACTGAATATTT<br>CCACTCTTTGGGTTGGCATCCATACTTTGTTTCTTCTTTCGATAAGCCA<br>ATCATGCAAGTCCACGAAGAAATGGCCAAAACTATGGACACCGTTTTC<br>ACTGAAATCAAGGACATTCGTGAAAAGGCTGTTCAACAAACTAACGA<br>AGAAATCACTAGACCATTGTGGCCAATGATTGTTTTAAGATCCCCAAA<br>GGGTTGGACTGGTCCAAAGACCTGGGATGACAAGCCAATCGAAAACT<br>CCTTCAGAGCCCATCAAATCCCAATCCCAGCTGACCAAAACCATCCTG<br>AATATATTCCACAATTAGTTGACTGGTTACAATCTTACAAGCCAGATGA<br>ATTATTTGACGAAAATGGACAATTGACCCAATCCATACAAGAAGTGCT

	ACCAAAGAAGGAATTGAGAATGGCTAATAACTCTGTTACCAACGCCG GTATCATTAAACCATTGATCTTGCCAGATATTGACAATTATTTGGTCGA AAACAACCAACCAGGTAACAACTTAGCTCAAGATGCTATTTTGTTGGG TGACTACTTGAGAGACATCATAAAGTTGAACCCAACTAATTTCAGAGG TTTCGGTCCAGATGAAACCGCCTCAAATAGATTCCAAGACATCTTCGA GACAACTAACAGACAATGGTTGTTGCCCATCAAGGAACCAAACGATC AATTCATGGCCCCAGAAGGTAGAATTATCGACTCCATGTTGTCTGAAC ACTACGATGAAGGTATGTTGGAAGCCTACACTTTGACTGGTAGACACG GTTTCTTTGCTTCCTACGAAGTTTTATCCGGGAAGTTGACGACATGAT TGTCCAACATTTCAAATGGTTAAACCACTCGCACGATGTCAGTTGGAG AAAGGATGTTCCAGCTTTGAACATTATTGCCGATTCTACTGTTTTCCAA CAAGACCACAATGGTTACTCTCACCAAGATCCAGGTGTCACCACTATG TTGTACGAAAAACAGCCAGACTTCATCCGTGAATTTTTCCCAGCTGAC GCCAACTCCCTGGTCGCTACTTTCGAACATGCTGCTCAAGCTACCCAA CAAATCAACTACATTGTTGCTTCCAAGCACCCAAGATTGCAATGGTTC TCTCCAACTGAAGCTAAGCAATTGGTTACTCAAGGTTTGCGTGTCATT GACTGGGCTTCCACCGATAAGGGTGAAAAGCCAGACATTATCATCGCA TCTGCTGGTTCCGAACCAACGACCGAATCTTTGGCTGCTATTCAAATT TTGCACGAACACATTCCATCTCTTAAGATCCGTTACATCAACGTCCTGG ACTTATTCAAGTTGAGAGCTGATGCCAACTACGGTTTGTCTGATGATG AATTTGACGCATACTTCACCACCGACACTCCAGTTTTGTTCGCTTTCCA CGGTTACGAACCTATGATCGAATCTATTTTCTTCAAGAGACATAACCAC CACTTGGCCGTCCACGGTTACAGAGAAGTTGGTGACATCACTACCCC ATTCGATATGAGAGTTTTAAATAAGATTGACAGATTCAACTTGGTCAA GGCTGCCATCAACTTGTTACCAGAAAACATCAGAACCAAGCAAGCCG CTCTCGTTCAAGAAATGACTGATAAATTGGACTTGCATGTTGCCTACA CCAGATCCAAGGGTACTGATTTGCCCGAAGTTGAAGACTGGAGATGG CAACCATTGAAGTAA
pta	ATGAAGTTGATGGAAAACATCTTCGGACTGGCTAAGGCTGACAAGAA GAAGATTGTCTTAGCTGAAGGTGAAGAAGAGCGTAACATTCGTGCTT CCGAAGAAATCATCAGAGATGGTATTGCTGACATTATCTTGGTCGGTTC TGAATCCGTTATCAAGGAAAACGCCGCTAAGTTCGGTGTTAACTTGGC TGGTGTCGAAATTGTTGACCCAGAAACTTCTTCTAAGACCGCCGGTTA TGCTAACGCTTTCTACGAAATTAGAAAGAACAAGGGTGTTACTTTGGA AAAGGCTGATAAGATCGTTAGAGATCCTATCTACTTCGCTACCATGATG GTTAAGTTGGGTGACGCTGATGGTTTGGTTTCCGGTGCCATTCACACT ACTGGTGATTTGTTAAGACCAGGCTTGCAAATCGTCAAGACTGTCCCA GGTGCTAGTGTTGTCTCTTCCGTTTTCTTAATGTCTGTTCCAGATTGTG AATACGGTGAAGATGGTTTCTTGTTATTTGCCGACTGTGCTGTCAACG TCTGTCCAACTGCTGAAGAATTATCTTCAATTGCCATCACCACTGCCG AAACCGCCAAGAACTTGTGTAAGATTGAACCAAGAGTTGCTATGTTGT CTTTCTCTACTATGGGTTCTGCTTCCCACGAATTGGTTGACAAAGTTAC CAAGGCCACCAAGTTGGCTAAAGAAGCTAGACCAGACTTGGACATTG ACGGTGAACTACAATTGGATGCTTCTTTGGTCAAAAAAGTGGCAGAC

	TTGAAGGCTCCAGGTTCCAAGGTTGCTGGTAAGGCTAATGTTTTGATT<br>TTCCCAGACATCCAAGCTGGTAACATTGGTTACAAGCTAGTTCAAAGA<br>TTCGCCAAAGCTGAAGCTATCGGTCCAATCTGCCAAGGTTTTGCTAAG<br>CCAATCAACGATTTGAGCAGAGGTTGTTCTGTCGATGACATCGTCAAG<br>GTTGTTGCTGTCACCGCCGTCCAAGCCCAAGCTCAAGGTTAA

Table S2. Primers used in this study

**Primers**	**Sequences (5' to 3')**
IoGlyR-GF	CGCGGATCCATGTCATTCTGGGATACGGGGAA
IoGlyR-GR	GCAAGCTTTTACAGATGCTGAAAGAGGCC
IoGlyR-VF	CTTTCAGCATCTGTAAAAGCTTGCGGCCGCACTCGAG
IoGlyR-VR	CAGAATGACATGGATCCGCGACCCATTTG
AtGlyR-GF	CGGATCCATGGAAGTTGGTTTCTTAGGTTTAG
AtGlyR-GR	GCCGCAAGCTTTATTCTCTAGAAAATTTAACAGCTTCAATAAC
AtGlyR-VF	TTCTAGAGAATAAAGCTTGCGGCCGCACTCGAG
AtGlyR-VR	CCAACTTCCATGGATCCGCGACCCATTTG
p415-IG-GF	TCTCGTTCATCTTATTCTTTAGCCAAGCGGCCGCTTTGAAACATCATG AAAACTGTTTC
p415-IG-GR	AACAAAAGCTGGAGCTCCACCGCGGGAAACTTAAGAATTCAATTAA CTACACAG
TEF1-F	CGAATTGTTTGAAACATCATGAAAACTGTTTCACC
TEF1-R	GTGTGTAGACCATTGTGATATATAAGTTAGATTTGTCTGTTGAAGG
ICL-F	TATCACAATGGTCTACACACAAATCAATTTAGAC
ICL-R	CGCCTCAGAATCTATTTGAATTGCTCTTCAGTGACA
3767-F	TTCAAATAGATTCTGAGGCGAACTATAGAATG
3767-R	GAACAAAAGCTGGAAACTTAAGAATTCAATTAACTACACAGTA
414-ICL-VF	CTTAAGTTTCCAGCTTTTGTTCCCTTTAGTGAG
414-ICL-VR	TTCATGATGTTTCAAACAATTCGCCCTATAGTGAGTCG
ICL-check-R	GCAATAATAGGTCTCAAGAAATCGG
glyR-check-R	CCATAATCATATTAACAATTAATTTCATTTTAGC
EG2-gRNA-F	GGTCTCATGCATAAGGCCTGGGTATACATCG
EG2-gRNA-R	GGTCTCGAAACCGATGTATACCCAGGCCTTA
EG2-LHR-F	CAATTTTCAAATAAAGTAAAGAAATAGATTTTCTG
EG2-LHR-R	GATGTTTCAAATAGGTGGTAAAACCAAGAGAGAAAAG
EG2-GF	GGTTTTACCACCTATTTGAAACATCATGAAAACTGTTTCA
EG2-GR	CAATTTAATCTACATACATTGGCTAAAGAATAAGATGAACGAGAAG
EG2-RHR-F	CTTTAGCCAATGTATGTAGATTAAATTGTCTACATATAAAATGATATGG
EG2-RHR-R	CATATAAACATATTCATATAGAGCAAGTGACGT
MLS-check-F	GATTTCCTCGGATAAGGGGC
MLS-check-R	GACCAAGTTGGAGCACAAGA
p414-XI-VF	GTTTAAACCAGCTTTTGTTCCCTTTAGTGAGGGT
p414-XI-VR	CGCCAATTCGCCCTATAGTGAGTCG
INO1-F	GGGCGAATTGCAACGGTGTAATCAGAGAG
INO1-R	AACACAACATTATATTCGTTTTTTTAGTTTGCTCTG
xks1-F	GCAAACTAAAAAAACGAATATAATGTTGTGTTCAGTAATTCAG
xks1-R	CTCGAATATTAGATGAGAGTCTTTTCCAGT
PFK1-F	GGAAAAGACTCTCATCTAATATTCGAGAAGGTTTCTACTG

PFK1-R	GGAACAAAAGCTGCCGTCTATTGTTCTCCTTCC
p414-xks-VF	CAATAGACGGCAGCTTTTGTTCCCTTTAGTGAGG
p414-xks-VR	CACCGTTGCAATTCGCCCTATAGTGAGTCG
GPM-F	CGAATTGCGAAAAATGCACCACACCCG
GPM-R	CAATGTTCATTTTGTGTGTGTGTTTTAAGATATACAATTG
xpk-F	CTTAAAACACACACACAAAATGAACATTGATTCTACCG
xpk-R	TGTTTCACCTTTACTTCAATGGTTGCCATC
MDH1-F	GGCAACCATTGAAGTAAAGGTGAAACACAACAACCTC
MDH1-R	GGGAACAAAAGCTGCGTACAGGGTTATAAAGTTGTTCAGAG
p414-xpk-VF	CCTGTACGCAGCTTTTGTTCCCTTTAGTGAG
p414-xpk-VR	GGTGCATTTTTCGCAATTCGCCCTATAGTGAGTCG
FBA1-F	CTATAGGGCGAATTGATGCCATATTGTATGTGTATTG
FBA1-R	CCATCAACTTCATTGTGTTTGTGTTGGGGGT
pta-F	CACAAACACAATGAAGTTGATGGAAAACATCTTCG
pta-R	CTAATCCACTTAACCTTGAGCTTGGGCTTG
5025-F	GCTCAAGGTTAAGTGGATTAGGTTACTGCTC
5025-R	CTCGAGCGGATAGATCGCGGATCCGGGTAAGAATAAAGGAAAAGGC
p414-pta-VF	GGATCCGCGATCTATCCGCTCGAGCAGCTTTTGTTCCCTTTAGTGAG
p414-pta-VR	GGCATCAATTCGCCCTATAGTGAGTCG
xyl-ENO2t-F	CGTACCGGTGAGATCTATTG
xyl-ENO2t-R	ACCGTTAGCCTCCTCTACAGGTGTATGTACCTGTCCCGCTGGAACAT GTTTGTACAAAT
xyl-A-F	TCTAACCTAAGGACTTAAATATTTGTACAAACATGTTCCAGCGGGAC AGGTACATACAC
xyl-A-R	TGCTGTTGCTGTGCAAGAGCAATTTTCTCTCTGATTACACCGTTGGT TTAAACTTTTTA
xyl-B-F	TCTAAATATGTCCCATCAGCTGGAAGGAGAACAATAGACGGCGAAA AATGCACCACACC
xyl-B-R	AGGGAACAAAAGCTGGAGCTCCACCGCGGTGGCGGCCGCCGTACA GGGTTATAAAGTTG
xyl-FBA-F	CCCTGTACGATGCCATATTGTATGTGTATTGTATTAAGTG
xyl-5025-R	CCTCACTAAAGGGAACAAAAGCTGGAGCTCCACCGCGGTGGCCTC GAGCGGATAGATCG
**qPCR-alg-F**	TCTTCCTCAGATGCAACTTCC
**qPCR-alg-R**	CTGCCATTTGCATGAGTTTCC
qpcr-XI-F	GGTTCTATTGATGCTAATAGAGGTG
qpcr-XI-R	GCTCTAGCCATAGCATCCATACC
qpcr-xks-F	CCGCATTATCACACAAAGAAGG
qpcr-xks-R	TGCAAATGCTACAGAGCTCAC
qpcr-xpk-F	CATGTTCTACATTGAAGGTCCAGG
qpcr-xpk-R	GCAATCAAGTCAGGGTTATCC
qpcr-pta-F	GCTAAGTTCGGTGTTAACTTGG
qpcr-pta-R	CTTGACGATTTGCAAGCCTG

qpcr-XR-F	CTAAGCTGATCGAGTACGTGCAG
qpcr-XR-R	CTTATCGGCGATAGACTTGATGGT
qpcr-XDH-F	GCAGTGAAGTGACTAATCTGAAGG
qpcr-XDH-R	GGATTTGGCTCATCGGGATTCA

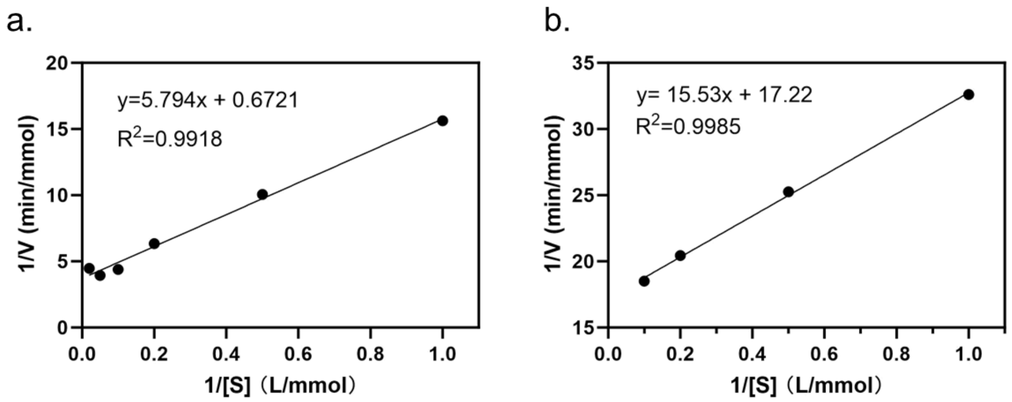

Figure S1. Double reciprocal plot of glyoxylate reductase on glyoxylic acid. (a) AtGLYR double reciprocal chart; (b) IoGLYR double reciprocal chart
